# OCT and OCT Angiography Update: Clinical Application to Age-Related Macular Degeneration, Central Serous Chorioretinopathy, Macular Telangiectasia, and Diabetic Retinopathy

**DOI:** 10.3390/diagnostics13020232

**Published:** 2023-01-08

**Authors:** Lyvia Zhang, Elon H. C. Van Dijk, Enrico Borrelli, Serena Fragiotta, Mark P. Breazzano

**Affiliations:** 1 Department of Ophthalmology & Visual Sciences, State University of New York Upstate Medical University, Syracuse, NY 13210, USA; 2 Leiden University Medical Center, 2333 Leiden, The Netherlands; 3 Ophthalmology Department, San Raffaele University Hospital, 20132 Milan, Italy; 4 Ophthalmology Unit, Department NESMOS, S. Andrea Hospital, University of Rome “La Sapienza”, 00189 Rome, Italy; 5 Retina-Vitreous Surgeons of Central New York, Liverpool, NY 13088, USA

**Keywords:** OCT, OCT angiography, age-related macular degeneration, diabetic retinopathy, central serous chorioretinopathy, macular telangiectasia

## Abstract

Similar to ultrasound adapting soundwaves to depict the inner structures and tissues, optical coherence tomography (OCT) utilizes low coherence light waves to assess characteristics in the eye. Compared to the previous gold standard diagnostic imaging fluorescein angiography, OCT is a noninvasive imaging modality that generates images of ocular tissues at a rapid speed. Two commonly used iterations of OCT include spectral-domain (SD) and swept-source (SS). Each comes with different wavelengths and tissue penetration capacities. OCT angiography (OCTA) is a functional extension of the OCT. It generates a large number of pixels to capture the tissue and underlying blood flow. This allows OCTA to measure ischemia and demarcation of the vasculature in a wide range of conditions. This review focused on the study of four commonly encountered diseases involving the retina including age-related macular degeneration (AMD), diabetic retinopathy (DR), central serous chorioretinopathy (CSC), and macular telangiectasia (MacTel). Modern imaging techniques including SD-OCT, TD-OCT, SS-OCT, and OCTA assist with understanding the disease pathogenesis and natural history of disease progression, in addition to routine diagnosis and management in the clinical setting. Finally, this review compares each imaging technique’s limitations and potential refinements.

## 1. Introduction

As a noninvasive imaging modality, optical coherence tomography (OCT) scanning has been used to reconstruct axial A-scan, cross-sectional B-scan, and three-dimensional images through light waves. Spectral-domain OCT (SD-OCT) is characterized by an enhanced sensitivity and signal-to-noise ratio. The OCT imaging speed ranges from 25,000 to 85,000 A-scans per second, making it widely used in diagnosing and monitoring eye disease. Swept-source OCT (SS-OCT) is a newer variation of Fourier-domain OCT. SD-OCT uses wavelengths of ~800 nm, while SS-OCT uses a longer light source of approximately 1000 nm [1,2]. Compared to SD-OCT, SS-OCT scans across a narrower spectrum of wavelengths using a center light source at 1 µm [3]. The longer wavelength offers SS-OCT a deeper penetration of tissue and subsequently more enhanced visualization of the choroid [4]. Both imaging techniques can be limited by media opacities such as either dense cataract or vitreous hemorrhage.

OCT angiography (OCTA) has recently emerged as a non-invasive modality for capturing blood flow using repeated B-scans [5]. The B-scans represent the dynamic blood flow while the surrounding blood vessels and tissues remain still. Therefore, compared to other types of OCT, OCTA can identify microvascular morphology and perfusion of various retinal and choroidal diseases. This review focused on four commonly encountered retinal diseases including age-related macular degeneration (AMD), diabetic retinopathy (DR), central serous chorioretinopathy (CSC), and macular telangiectasia (MacTel). The objective is to demonstrate how these modern imaging modalities further shape clinical diagnosis and management in ophthalmology.

## 2. Age-Related Macular Degeneration

### 2.1. Background

Age-related macular degeneration (AMD) is a degenerative retinal disease that mainly affects the fovea and parafovea. Patients may experience visual disturbances, including dark spots appearing in the center of the visual field, creating challenges in daily living. AMD is one of the leading causes of blindness, and approximately 8.7% of the worldwide population suffers from it. The predicted disease population will be approximately 288 million in 2040 [6]. 

There are two subtypes of AMD, classically “wet” and “dry” forms, with recently further nuance into “exudative” and “non-exudative” as well as “neovascular” and “non-neovascular” classifications, respectively [7]. However, most clinicians employ a classification that divides AMD into three stages—early, intermediate, and late—based on the size and extent of drusen as well as any related complications [8]. Of note, the late stage of the disease is characterized by the presence of macular neovascularization or geographic atrophy (GA).

### 2.2. AMD on OCT and OCTA

Drusen are the hallmark of AMD and are typically located between the retinal pigment epithelium (RPE) and Bruch’s membrane. Patients are often asymptomatic in the early stage of the disease [8]. The presence of crystalline deposits corresponding to hyperreflectivity by OCT is consistent with a histologic finding of cholesterol crystals in the subretinal RPE layer, and is highly associated with AMD. These hyperreflective cholesterol crystals can be stratified as parallel, highly reflective structures in the subretinal space, also known as “onion sign”, a signature feature of neovascular AMD observed by SD-OCT [9]. Conversely, its presence in non-neovascular AMD is used as an imaging biomarker for a progressive complication of late-stage AMD. Of note, the high reflectivity of such structures can produce peculiar reflection artifacts visible with both SD-OCT and OCTA [10].

A combination of imaging modalities was adapted to detect early AMD. The OCT B-scans detect the presence of drusen, which are then classified as AMD or normal cases. The region of interest on the B-scan can be further analyzed using fundus photography [11,12,13]. OCT, then, is suitable for the detection of drusen and the early screening for AMD. OCTA has largely been employed to study patients with drusen (i.e., early/intermediate AMD). These studies have demonstrated that the choriocapillaris is impaired in patients with early/intermediate AMD [14,15].

The dysfunction of the unit comprised of photoreceptors in the outer retina, RPE, Bruch’s membrane, and choriocapillaris may eventually lead to choroidal (i.e., type 1 and 2) or intraretinal (i.e., type 3) neovascularization. Poorly formed vessels develop within the proliferation of fibrous tissue in the “wet” form of AMD [16]. Over time, these vessels become leaky and may cause intraretinal and/or subretinal and/or sub-RPE exudation, resulting in decreased visual acuity. Moreover, the sensitivity and acquisition speed of SD-OCT allow it to identify the neurosensory or RPE elevation with associated neovascularization [17,18]. Therefore, OCT has been used to precisely analyze the intraretinal and subretinal fluid and retinal thickness, making OCT a critical imaging tool for managing AMD [18].

The atrophy of the RPE is also known as GA. It enlarges over time and becomes more prominent in the advanced stage of AMD [19]. GA can be found using OCT with features of ellipsoid zone disruption, refractile drusen, and hyperreflective foci [13,19]. This area can be quantified on various imaging modalities including SD-OCT with fundus autofluorescence (FAF), color fundus photography, and near-infrared reflectance (NIR) imaging [20]. Furthermore, SD-OCT can be used to identify GA precursor regions and associated local photoreceptor changes and drusen accumulation up to 4 years prior to the onset of GA. These identifiable clinical endpoints offer potential drug therapy targets in trials [21]. Additionally, the baseline and mean annual enlargement rates of GA also provide quantitative metrics for staging and monitoring the disease progression. The clinical value in utilizing OCT as part of routine care with multimodal imaging in AMD cannot be understated. Finally, OCTA also reveals biomarkers of GA enlargement over time, as previous studies have shown that choriocapillaris hypoperfusion is directly associated with atrophy growth [22].

Intraretinal and/or subretinal fluid may complicate various ocular diseases including AMD. It has several underlying mechanisms. For example, in diabetic retinopathy, the intraretinal vessel leakage results in fluid accumulation within the neurosensory retina. Conversely, intraretinal and subretinal fluid in AMD results from exudation secondary to neovascularization [23,24]. OCTA assesses structural changes in vessels and differentiates choroidal neovascularization in “active” and “silent” leakage [25]. Furthermore, OCTA volume rendering [26] demonstrates the intraretinal origin (rather than choroidal) of macular neovascularization type 3 with exudative AMD [26,27]. This enhances the OCTA visualization of deep retinal tissues.

Intraretinal and/or subretinal fluid in the context of AMD may also be present in the absence of neovascularization. SS-OCTA and dense B-scan OCTA can reveal the presence of fluid in the setting of non-neovascular exudation. This novel phenotype of AMD suggests that intraretinal fluid accumulation is not a definitive indicator of active leakage [28]. Similarly, pseudocysts in atrophic AMD are used to explain the cystoid spaces of the inner nuclear layer in the absence of exudation [23]. The nuances of the clinical imaging findings help us understand each subtype of AMD. Moreover, OCT also provides a predictive value of anti-vascular endothelial growth factor (VEGF) therapy in exudative and nonexudative AMD and their potential outcomes [25,29]. OCTA can provide additional benefit in differentiating exudative, non-neovascular cases as well as non-exudative neovascular cases by directly visualizing the aberrant blood vessel flow.

Current treatment for AMD is a combination of lifestyle/dieting modification, multi-vitamin supplementation, smoking cessation, and anti-VEGF [30,31,32,33]. Early recognition of neovascular AMD on imaging allows for prompt management and delaying the progression of late-stage complications. 

## 3. Central Serous Chorioretinopathy

### 3.1. Background

Central serous chorioretinopathy (CSC) is also known as central recurrent retinitis or idiopathic central serous chorioretinopathy. CSC is a common maculopathy that mainly affects men aged 20–50, with a presentation of distorted central vision, micropsia, metamorphopsia, paracentral scotoma, and decreased color vision or near vision. This condition is typically classified as acute or chronic as exudation with CSC may often persist and/or recur. Although the exact underlying cause is poorly understood, it is associated with hypercortisolism, caused either by endogenous or exogenous cortisol [34,35]. Hypercortisolism has been hypothesized to lead to vasospasm and permeability of the choroidal vessels. The hyperpermeable vessels cause fluid accumulation in the sub-retinal space, with the potential consequence of serous RPE detachment. Additionally, the fluid accumulation in the sub-retinal space spreads out the photoreceptors, leading to classic signs of micropsia and changes in color vision. In addition to hypercortisolism, other studies have found that CSC is associated with type A personality [36], which ultimately leads to stress-induced hypercortisolism [37], contributing to the development of CSC. Other associations include obstructive sleep apnea [38], helicobacter pylori infection caused abdominal pain [39], Cushing syndrome including steroid use, and the endogenous overproduction of steroids [40]. Several genes have also been linked to CSC [41,42,43,44]. 

CSC generally has a good prognosis, with a reasonable chance of complete subretinal fluid resolution within three months after diagnosis [45,46]. When CSC with RPE abnormalities persist longer than six months, it becomes chronic CSC [47]. In a prospective case control study, among 50 patients with acute CSC, 52% of them had used steroids in the prior month. The study also demonstrated a favorable outcome in patients with distorted vision after discontinuation or tapering steroids [48]. Therefore, early detection of CSC and the timely discontinuation of steroids lead to a faster resolution of symptoms. Another study showed that half of the study patients experienced a recurrence of symptoms [49], and 15% of patients showed persistent accumulation of serous retinal fluid for longer than six months [50]. Therefore, regular follow-up along with imaging is essential in the management of CSC.

### 3.2. CSC on OCT

Traditional imaging techniques such as fluorescein angiography (FA) and indocyanine green angiography (ICGA) are commonly used in the diagnosis of CSC. SD-OCT is a reliable imaging of choice for evaluating and monitoring the progression of CSC. Enhanced depth imaging (EDI-OCT) and SS-OCT can be used concurrently to visualize the structure and thickness of the choroid, providing a more in-depth image of the CSC [51]. SS-OCT with relatively high penetration of light waves facilitates the visualization of widefield changes in choroidal thickness, the dilated outer choroidal vessels with choriocapillaris compression, or thinning. 

The approximate average subfoveal choroidal thickness threshold ranges from 300 μm to 395 μm [51,52,53]. However, the choroid is usually thicker in CSCR and other diseases that are part of the pachychoroid disease spectrum. Moreover, thickening of the inner choroidal blood vessels and subsequent thinning of the choriocapillaris can also be easily appreciated on OCT [52,54]. Of note, a thinning of the CC was demonstrated to be associated with a greater likelihood of the development of macular complications (i.e., neovascularization or RPE atrophy) within 3 years [55]. The qualitative and quantitative measurements of the choroidal thickness come with challenges, and many factors should be considered, including imaging artifacts and pathologic effects. The subfoveal choroid of CSCR with choroidal vascular hyperpermeability was statistically thicker (410 ± 92 μm) compared to fellow eyes in CSCR without choroidal vascular permeability. The EDI-OCT measures the choroidal thickness in a noninvasive fashion through a better delineation of the choroid–scleral junction at the posterior pole. It may be conceivable that the focal or diffuse choroidal venous dilation seen on OCT may be associated with choroidal vascular hyperpermeability [56]. Additionally, EDI-OCT also demonstrated the increased thickness of the Haller layer in CSC compared to fellow eyes and in the normal controls [57]. OCT has also been used to detect serous subretinal fluid, the hallmark finding of CSC, which is sometimes accompanied by intraretinal fluid. Moreover, OCT may reveal RPE alterations and detachments within and outside the neurosensory serous detachment as well as disruptions in the integrity of the outer retinal layers [58]. With a high recurrence rate of 40–50% in patients with CSC, chronic CSC differentiates from acute CSCR. In chronic CSC, a “double-layer sign” is more often present, which is formed between the shallow irregular RPE detached from the retina and Bruch’s membrane and could be indicative of a choroidal neovascularization [59]. 

### 3.3. CSC on OCTA

Previously, the use of ICGA showed that choroidal vascular flow combined with RPE detachment contributed to choroidal vascular hyperpermeability. The introduction of OCTA as a newer, noninvasive imaging modality improved the understanding of the pathogenesis of CSC [60]. The innermost layer of the choroid, the choriocapillaris, supplies nutrients to the RPE and photoreceptors [61]. With SS-OCTA, potential neovascularization of the choriocapillaris in CSC is often well-appreciated, more frequently showing a larger low flow area than in the control eyes. Additionally, the abnormal choroidal vasculature has been found to reduce the choriocapillaris function in CSC given the greater mean flow void area and choroidal capillary bed ratio [62]. Apart from visualization of choroid vessels, OCTA is also used to assess the choriocapillaris blood flow quantitatively through post-processing using dedicated software for image manipulation. In a case-control study, the total area of flow signal voids was quantified at baseline and followed-up at 3 and 6 months. The outcome suggests that the ischemia of the choroidal bed results from abnormal and dilated choroidal vessels that may compress the overlying choriocapillaris. The flow deficits explain the later development of visual distortion and micropsia in CSCR [63]. Finally, a previous OCTA report showed that a number of chronic CSCR patients may be featured by changes in the retinal vessels [64].

Most patients with CSCR achieve complete resolution of visual disturbances within 2–3 months, without the need for treatment. In those with chronic or recurrent flares of CSCR, reduced settings of photodynamic therapy with verteporfin (vPDT) have been found to be the treatment of choice [45,65,66,67,68]. Additionally, OCTA facilitates the treatment of vPDT in chronic CSCR. Eleven review studies demonstrated the quantitative and qualitative measures of choriocapillaris changes after vPDT treatment. The assessments included the variability of flow pattern, density of choroid vessels, and choroidal perfusion changes. OCTA is essential in treatment therapy adjustment and the anatomical and functional monitoring of choriocapillaris [69]. 

**Figure 1:**
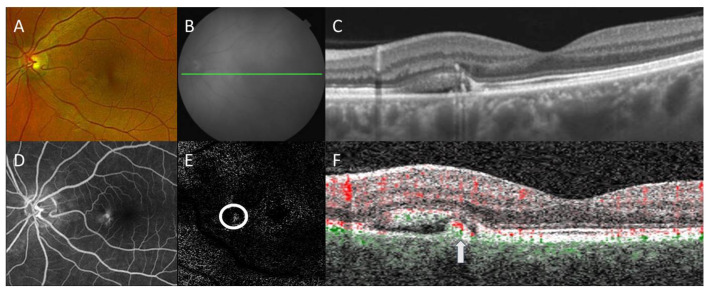
Multimodal imaging of central serous chorioretinopathy with secondary choroidal neovascularization. Color fundus photography (**A**) shows a retinal pigment epithelial (RPE) detachment with surrounding subretinal fluid nasal to the fovea. Corresponding *en face* near-infrared reflectance (**B**) with B-scan (green line) by swept-source optical coherence tomography (OCT) demonstrates a thickened choroid with overlying pigment adjacent to the subretinal fluid (**C**). Late-phase fluorescein angiography (**D**) revealed corresponding hyperfluorescence without overt indication of choroidal neovascularization. *En face* OCT angiography (OCTA) with segmentation between the outer retina and RPE (**E**) revealed the focal area of the flow signal (circle), which on B-scan (green: choroidal flow, red: retinal flow) localizes (arrow) within the RPE detachment (**F**), consistent with secondary choroidal neovascularization. Projection artifacts were removed automatically for OCTA.

**Figure 2:**
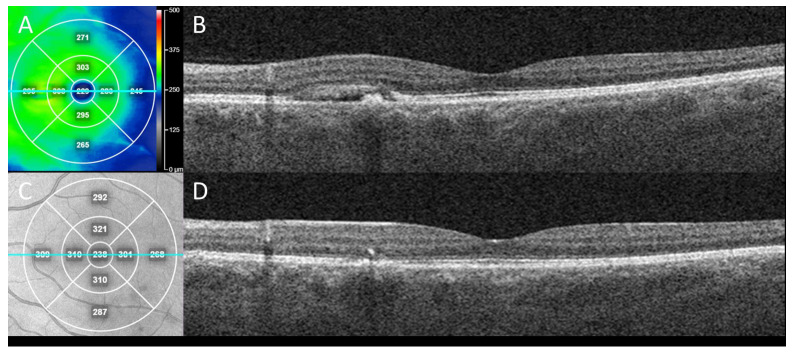
Clinical outcome from case in Figure 1 following treatment for CSCR with secondary neovascularization. *En face* swept-source OCT “heat” thickness map (**A**) with corresponding line (teal) OCT B-scan indicates subretinal fluid with RPE detachment in nasal macula of left eye at presentation (**B**) which alone could be mistaken for CSCR without neovascularization. This eye was treated with two sequential, intravitreal anti-vascular endothelial growth factor (VEGF) injections (bevacizumab), one month apart. Three months after initial presentation, *en face* near-infrared reflectance (**C**) with corresponding line (teal) OCT B-scan demonstrated sustained resolution of the subretinal fluid and collapse of the RPE detachment (**D**), consistent with response of exudation and neovascularization to anti-VEGF treatment.

OCTA can be instrumental for the identification of secondary neovascularization, which may occur in the minority of CSCR patients (Figure 1). In four patients with chronic CSCR who were offered sildenafil treatment, SS-OCTA showed a small amount of abnormal flow in the choroids with subretinal fluid leakage. Furthermore, OCTA also excludes choroidal neovascularization in patients who respond to treatment with improved visual acuity [70]. Thus, the use of OCTA for guiding treatments including intravitreal anti-VEGF is well-founded in many cases, appropriately impacting the management course when the possibility of secondary neovascularization may be ignored or missed (Figure 2). Patients were found to have an irregular, shallow PED with a double layer sign and flow signal on OCTA, consistent with macular neovascularization type 1 in 95% of the cases analyzed, suggesting the importance of the prompt recognition of this feature and the usefulness of confirmatory OCTA [71].

## 4. Macular Telangiectasia

### 4.1. Background

Macular telangiectasia (MacTel), also known as idiopathic macular telangiectasia, is a rare group of neurodegenerative diseases caused by loss of Müller cells with abnormal anastomoses of foveal and perifoveal capillaries. It results in the degeneration of the outer retinal layer and even the development of a macular hole in the advanced stage. It constitutes three subtypes, with type 2 (MacTel 2) the most common form presenting as a bilateral disease, having an association with diabetes mellitus and hypertension. MacTel 2 affects men and women equally, with a prevalence of 0.1% in patients aged 40 and older [72]. Although MacTel 2 was originally described by Gass [73] as a “zone of light gray discoloration” with vascular anastomoses in the central macula, the changes in the RPE result in retinal hyperplastic and stellate plaques [74]. With the emergence of multimodal imaging, the 2025 MacTel Study Project has established that the disease pathogenesis is caused by vascular abnormalities secondary to other changes of the macula. 

### 4.2. MacTel on OCT and OCTA

MacTel 2 can be divided into non-proliferative and proliferative stages, with neovascularization in the advanced disease. Based on the depth of the neovascularization into the outer and inner retina, proliferative MacTel can be further subdivided [75]. SD-OCT is used to characterize MacTel clinical features in various stages and establish the relationships between the imaging findings and the disease progression and associated visual acuity. In the non-proliferative stage of MacTel 2, a hyperreflective middle retinal layer from capillary leakage can be noted on imaging. Findings of an irregular fovea and hyperreflective RPE clumps are common in the advanced stage of MacTel 2 with poor vision [76]. This evidence indicates that SD-OCT is suitable for staging disease progression. 

FA is often considered to be the gold standard diagnostic tool for MacTel. It is useful in demonstrating intraretinal anastomoses. However, fluorescein leakage can interfere with the visualization of the underlying subretinal neovascularization. OCT assists in identifying increased retinal thickness along with subretinal neovascularization [77]. Therefore, FA and OCT are used in combination to characterize these complications throughout the tissue depth with greater clarity.

Normally, the central retinal artery and the short posterior ciliary arteries supply the retina. The central retinal artery and its branches travel alongside the optic nerve, supplying the inner retinal layer. In MacTel, an increased intervascular network with diminished capillaries and abnormal vessel anastomosis is noted on OCTA. The quantified superficial and deep retinal capillary density and branches are significantly reduced compared to healthy eyes [78,79]. This finding suggests that the abnormal anastomosis of vessels contributes to hypoperfusion of the retina, which may play a role in the disease progression. The ellipsoid zone (EZ), also known as the inner/outer segment of photoreceptors (IS/OS), and its destruction or loss is independent of severe visual impairment using SD-OCT. The perifoveal disturbance unaffecting the visual acuity explains this clinical finding in advanced disease [80]. Moreover, SD-OCT-facilitated OCTA has been used to study the relationship between EZ loss and the disease stages. Abnormal telangiectasia is found to be near the EZ peripherally in the early non-proliferative stage and moves centrally, overlapping with EZ loss, in advanced MacTel 2 [75]. This suggests a stepwise overlap progression of neovascularization and a linear correlation with EZ loss [75]. A persistent hyperreflective band with a progressive vascular pattern was also found in the outer retina on SD-OCT. This is evident in MacTel 2 with EZ loss in the temporal and the foveal region. It supports the neovascular pattern associated with advanced MacTel 2 [79]. 

OCTA has demonstrated utility in disease staging and its relationship to vascular changes for a variety of macular conditions [81]. Specifically, OCTA showed *de novo* genesis of retinal-choroidal anastomosis (RCA) in the absence of focal pigmentation or structural fluid exudation or previous subretinal neovascularization [82]. Furthermore, areas of RCA appear to colocalize with the outer retinal hyperreflective lesion (ORHL). This observation supports the hypothesis that new RCA formation precedes the findings of subretinal neovascularization. The emergence of RCA also followed the outer-retinal subsidence, demonstrated both with OCTA and OCT, consistent with the anatomic collapse and retinal cavitation after the loss of Müller cells and photoreceptors [82]. With the assistance of multimodal imaging including FA, SD- and SS-OCT, the application of OCTA has further improved the understanding of the disease process in MacTel.

## 5. Diabetic Retinopathy

### 5.1. Background 

Diabetic retinopathy (DR) is one of the leading causes of preventable blindness in patients aged 20 to 64 in the United States, with an increased risk in those with pre-existing type 1 or 2 diabetes [83]. It is more common in type 1 diabetes (40%) than type 2 (20%) [84]. Based on a survey by the World Health Organization, the approximate population with diabetes will increase from 171 million in 2000 to 693 million by 2045 [85,86]. Out of the macrovascular and microvascular complications, DR is the most common cause of blindness due to proliferative disease or macula edema [87]. Other ocular complications that result from diabetes include cataracts, glaucoma, cranial nerve palsies, corneal erosions, and ischemic optic neuropathy [88]. The pathogenesis of DR is poor glycemic control, which causes endothelial and pericytic damage. This process activates platelet adhesion and aggravates erythrocyte attachments, with subsequent lipid disturbance and abnormal blood viscosity. The inflammation from fibrin and platelet adhesion aggravates an increase in glycosylated aminoglycans, which further hypoperfuses the underlying retinal tissues, leading to neovascularization [89]. Other risk factors of DR include hyperlipidemia, microalbuminuria, and hypertension [87]. Therefore, early diagnosis and prompt blood pressure and glucose control significantly reduce diabetic complications [90]. 

### 5.2. DR on OCT and OCTA

There are two subtypes of DR: non-proliferative and proliferative. Based on the duration of diabetes and its severity, the overall understanding with research and the management of DR has improved using conventional imaging techniques including OCT and OCTA [91]. This review focuses on the clinical findings with these imaging modalities. 

Diabetes mellitus carries the risk of vision loss from two main causes: macular edema from capillary leakage and macular ischemia from capillary occlusion. The capillary plexus of the retina web is within the inner retina as well as the deep ganglion cell, inner plexiform layer, and deep inner nuclear layers [92]. The fovea within the ganglion cell layer is avascular, and its neovascularization is a pathognomonic finding in proliferative diabetic retinopathy. SD-OCT detects the disorganization of the inner retinal layers, which are also the biomarkers of centrally involved diabetic macular edema (DME) with decreased visual acuity [93]. Moreover, the extent of the disorganization of retinal inner layers (DRIL) positively correlates to visual acuity with decreased vision as the DRIL expands. This suggests that DRIL can be a reliable marker for visual changes in eyes with pre-existing DME [94,95,96]. 

The aminoglycans and inflammation alter the integrity of retinal vessels, leading to ischemia of superficial vessels and neovascularization, but also involving the choroid [97,98]. SD-OCT is used to measure the choroidal thickness, which normally ranges from 250 to 350 μm in healthy eyes [99]. A recent study used SS-OCT to map the choroidal thickness in the eyes of type 2 diabetic patients, demonstrating diffuse choroidal thinning on imaging. An ellipsoid pattern of choroidal thickness was found in all studied eyes, regardless of the history of diabetes [100]. 

Thinning of subfoveal choroidal thickness can be used as a biomarker preceding the development of diabetic retinopathy. This finding was evident in a recent meta-analysis showing that the choroid was markedly thinner in diabetic non-retinopathy eyes compared to healthy eyes [101]. Additionally, the choroid was thinner in DR compared to diabetic eyes without retinopathy [102]. The subtle changes in choroidal thickness differentiate early stage from more advanced DR on SS-OCT [103]. Some data have shown that in mild DR, the choroidal thickness increased slightly, and subsequently gradually decreased as DR worsened [104,105]. This observation explains the clinical variation in the choroidal thickness in the early stage of DR and suggests that a multimodal imaging approach may be essential for managing and monitoring diabetic retinopathy. 

DME arises from underlying ischemic blood vessels due to inflammation and hypoperfusion. The prolonged hyperglycemia generates an off-balance of decreased retinal oxygen supply and increased oxygen demand from vasodilation [106,107,108]. This disruption results in leaky vessels from the increased hydrostatic pressure, causing fluid accumulation in the macula. The underlying angiogenesis and inflammation are both involved in the development of DME. However, the causal relationship remains unclarified: it is therefore unknown whether angiogenesis is a cause or a result of the inflammatory process [106]. The advancement of OCT has led to a more specific classification of DME based on its imaging findings. The four main types of DME on OCT are diffuse retinal thickening, cystoid macular edema, serous retinal detachment (SRD), and mixed type [109]. The variation of inflammation was found in each subtype, requiring type-specific treatment. The SRD type has a poor prognosis for vision loss. Hence, higher concentrations of inflammatory cytokines such as IL-6 in the aqueous humor and vitreous have promoted various clinical trials of drug therapies targeting inflammation, specifically in the SRD type [109].

DME can also be subdivided into center-involving and non-center involving. The retinal thickening from center-involving DME is the most common cause of vision loss in the diabetic population [110]. The foveal avascular zone (FAZ) is located anatomically in the foveal center, physiologically lacking blood supply. The RPE pump is the only mechanism to reabsorb extracellular fluid at the FAZ level [107], which explains the predilection for fluid collection in this anatomical region. 

OCTA reveals a series of biomarkers for DR and DME. Eyes with greater FAZ area and lower vessel density in the deep capillary plexus are associated with a higher risk of progression of diabetic retinopathy. Furthermore, the vessel density of the superficial capillary plexus predicts the formation of DME [111,112,113,114]. In addition, OCTA shows that progressive decreasing in the vessel density of the macula correlates with worsening diabetic retinopathy. Along with vasculature changes, both non-proliferative DR and DR without clinical symptoms show a significant loss of focal volume of the ganglion cell complex. Therefore, OCTA has the potential to be a screening test for detecting vascular and neuronal damage in preclinical DR [113,115,116,117].

Fluorescein angiography (FA) is the gold standard of imaging for the diagnosis of diabetic macular ischemia, which is the occlusion and atrophy of retinal capillaries in the macula [118,119,120]. However, FA is a two-dimensional and invasive procedure compared to OCTA. OCTA also has the potential to explore the microvascular changes at the anatomic level, providing insights into the pathogenesis of diabetic retinopathy [111,118]. SS-OCTA is often more suitable in certain cases of dense or widespread retinal hemorrhage. Visualization with fluorescein can be obscured by overlying hemorrhage, as seen in a case of a retinal macroaneurysm; this shortcoming can be overcome by more costly ICGA or alternatively SS-OCTA, which both utilize deeper penetration from longer wavelengths of light [121]. Additionally, several OCTA studies have assessed macular ischemia in patients with diabetes. All of these studies have shown that macular hypoperfusion is an early finding in patients with diabetes, even in the absence of DR [122,123]. 

## 6. Discussion

### 6.1. Limitations of SS-OCT and SD-OCT

A prominent limitation in modern ophthalmic imaging is the presence of artifacts. Among these is the reflection artifact, evident with both SS- and SD-OCT. The OCT technique generates images based on the backscattered light from tissue, and a strong scattering leads to artifacts when the fluctuating signal supersedes the wavelength range the machine can detect [10]. For instance, cholesterol crystals are seen as hyperreflective signals on OCT and its planar reflection may generate artifacts [10]. 

### 6.2. Limitations of OCTA

OCTA images blood flow and vasculature details using motion contrast in the absence of intravenous fluorescein. Although OCTA is helpful in evaluating these characteristics in the retina and choroid including choriocapillaris vessel density, artifacts may affect the interpretability of OCTA. One type of artifact is a segmentation defect that presents altered vessel density and locations in erroneous places [4]. The OCTA generates qualitative slabs, demonstrating dynamic changes in the blood flows. Depending on the signal and noise of each region, some volumes may show a false flow [4]. 

Another type of artifact is a projection artifact. As light passes through a vessel, it is reflected, refracted, or absorbed. When light enters the tissue underneath the vessel, it is also reflected back to the OCT. This can create a false pattern of vessels, also known as projection artifacts, commonly in the retinal layer, choroid, and even sclera [4]. Algorithms have been incorporated in the software of some OCTA devices to counteract these effects [74].

Aside from artifacts, OCTA also lacks the ability to capture the plasma flow. Therefore, it has not been used to diagnose leakage points [124]. Currently, it is used in conjunction with other imaging modalities to aid in the diagnosis of ocular diseases. 

### 6.3. Future of Imaging

The concept of artificial intelligence (AI) was first introduced by McCarthy et al. during the Dartmouth Summer Research Project in 1955 [125]. Since then, many medical specialties have adopted its application in facilitating the diagnosis and management of diseases [126]. Ophthalmology is one of the leading specialties that expands the functions of OCT using AI. Currently, Altris (Chicago, IL, USA) [127] AI is available commercially. Different algorithms serve varying purposes in early screening and detecting retinal diseases. For instance, AI adopts a learning-based approach algorithm to differentiate the foreground and background objects in B-scans. Another learning model, the machine learning algorithm, is more commonly used with OCTA. By analyzing angiograms of single structural B-scans, AI recovers angiographic images and thus increases the available imaging data for OCTA quantification [128]. This approach can be particularly helpful in evaluating retinal microvasculature [129]. Furthermore, AI analyzes imaging patterns on OCT and provides predictable values in disease progression. Some algorithms recognize nuances in artifacts, and this makes the combination of AI and OCT capable of accurately detecting the conditions [128,130,131]. However, AI has several pitfalls that need to be considered. First, it requires a large volume of data as the reference and has yet to grow with a wider use of AI programs. AI also undermines the holistic approach of analyzing lesions on imaging compared to a skilled clinician, as AI often lacks the flexibility of recognizing subtle variations [130,132,133,134]. As AI algorithms and imaging techniques advance, there is an anticipation that AI will continuously translate structural data into functional information to optimize imaging interpretation [131].

## 7. Conclusions

OCT and OCTA have advanced in the last decade, and now they are widely used clinically to improve the efficacy of diagnosing and treating retinal disease. Moreover, these modalities have enhanced our understanding of disease processes and morphologic changes with excellent resolution. OCT and OCTA may have a complementary role in detecting pathologic microvascular modifications, neovascular complications, and offer predictive and prognostic insights for late-stage complications. Numerous OCT and OCTA biomarkers have been identified and characterized, helping in the refinement of AI algorithms and deep learning models. Although imaging comes with inherent artifacts, understanding the mechanism of artifacts, cooperating with AI algorithms, and integrating the judgement of experienced clinicians may all improve the application of multimodal imaging techniques.
